# A first genome assembly of the barley fungal pathogen *Pyrenophora teres *f. *teres*

**DOI:** 10.1186/gb-2010-11-11-r109

**Published:** 2010-11-10

**Authors:** Simon R Ellwood, Zhaohui Liu, Rob A Syme, Zhibing Lai, James K Hane, Felicity Keiper, Caroline S Moffat, Richard P Oliver, Timothy L Friesen

**Affiliations:** 1 Department of Environment and Agriculture, Curtin University, Kent Street, Bentley, Perth, Western Australia 6102, Australia; 2 Department of Plant Pathology, North Dakota State University, Fargo, North Dakota 58105, USA; 3 CSIRO Plant Industry, Centre for Environment and Life Sciences, Private Bag 5, Wembley, Western Australia 6913, Australia; 4 South Australian Research and Development Institute, Waite Institute, Adelaide, South Australia 5064, Australia; 5 Division of Health Sciences, Murdoch University, Murdoch Drive, Perth, Western Australia 6150, Australia; 6 USDA-ARS Cereal Crops Research Unit, Northern Crop Science Laboratory, 1307 18th Street North, Fargo, North Dakota 58105, USA

## Abstract

**Background:**

*Pyrenophora teres *f. *teres *is a necrotrophic fungal pathogen and the cause of one of barley's most important diseases, net form of net blotch. Here we report the first genome assembly for this species based solely on short Solexa sequencing reads of isolate 0-1. The assembly was validated by comparison to BAC sequences, ESTs, orthologous genes and by PCR, and complemented by cytogenetic karyotyping and the first genome-wide genetic map for *P*. *teres *f. *teres*.

**Results:**

The total assembly was 41.95 Mbp and contains 11,799 gene models of 50 amino acids or more. Comparison against two sequenced BACs showed that complex regions with a high GC content assembled effectively. Electrophoretic karyotyping showed distinct chromosomal polymorphisms between isolates 0-1 and 15A, and cytological karyotyping confirmed the presence of at least nine chromosomes. The genetic map spans 2477.7 cM and is composed of 243 markers in 25 linkage groups, and incorporates simple sequence repeat markers developed from the assembly. Among predicted genes, non-ribosomal peptide synthetases and efflux pumps in particular appear to have undergone a *P. teres *f. *teres*-specific expansion of non-orthologous gene families.

**Conclusions:**

This study demonstrates that paired-end Solexa sequencing can successfully capture coding regions of a filamentous fungal genome. The assembly contains a plethora of predicted genes that have been implicated in a necrotrophic lifestyle and pathogenicity and presents a significant resource for examining the bases for *P. teres *f. *teres *pathogenicity.

## Background

Net blotch of barley (*Hordeum vulgare*) is caused by *Pyrenophora teres *Drechsler (anamorph *Drechslera teres *[Sacc.] Shoem.). *P. teres *is an ascomycete within the class Dothideomycetes and order Pleosporales. This order contains plant pathogens responsible for many necrotrophic diseases in crops, including members of the genera *Ascochyta*, *Cochliobolus*, *Pyrenophora*, *Leptosphaeria *and *Stagonospora*. Net blotch is a major disease worldwide that causes barley yield losses of 10 to 40%, although complete loss can occur with susceptible cultivars in the absence of fungicide treatment [1]. In Australia the value of disease control is estimated at $246 million annually with average direct costs of $62 million annually, making it the country's most significant barley disease [2].

Net blotch exists in two morphologically indistinguishable but genetically differentiated forms: *P*. *teres *f. *teres *(net form of net blotch, NFNB) and *P*. *teres *f. *maculata *(spot form of net blotch, SFNB) [3,4]. These forms have been proposed as distinct species based on the divergence of *MAT *sequences in comparison to *Pyrenophora graminea *[4]. Additionally, it has been suggested that limited gene flow may occur between the two forms [5,6]. As their names indicate, the two forms show different disease symptoms. NFNB produces lattice-like symptoms, in which necrosis develops along leaf veins with occasional transverse striations. SFNB displays more discrete, rounded lesions, often surrounded by a chlorotic zone. NFNB and SFNB may both be present in the same region but with one form prevailing in individual locales. NFNB has historically been regarded as the more significant of the two diseases, but in recent years there have been reports of SFNB epidemics, notably in regions of Australia and Canada [7,8].

Only recently have researchers begun to focus on the molecular and genetic aspects of *P. teres *pathogenesis and host-pathogen interactions. NFNB is known to produce non-host selective low molecular weight compounds that cause chlorosis on barley leaves [9]. Both forms also produce phytotoxic proteinaceous effectors in culture [10,11]. It has been suggested that these effectors are responsible for the brown necrotic component of the disease symptoms on susceptible cultivars. Host resistance to *P. teres *appears to conform to the gene-for-gene model [12]. Both dominant and recessive resistance loci have been reported that are genetically distinct. These are host genotype, form, and isolate specific, and occur along with multigenic/quantitative resistance on each of the barley chromosomes [13,14].

Little is known at the molecular level about the mechanisms of *P*. *teres *pathogenicity, with neither the mechanism of virulence nor host resistance known. A genome assembly offers a powerful resource to assist the dissection of virulence mechanisms by providing suites of genetic markers to characterize and isolate genes associated with virulence and avirulence via map-based cloning. It also enables potential effector candidate genes to be identified from partially purified active fractions in conjunction with mass spectrometry peptide analysis. The sequencing and assembly of fungal genomes to date have relied primarily on Sanger sequencing with read lengths of 700 to 950 bp. Several newer sequencing technologies are now available that are orders of magnitude less expensive, although currently they exhibit shorter read lengths. These include Roche/454 pyrosequencing (400 to 500 bp) and Illumina/Solexa sequencing (currently up to 100 bp). Recent improvements, including paired-end sequencing (reads from each end of longer DNA fragments) and continuing increases in read lengths should make the *de novo *assembly of high quality eukaryotic genomes possible.

Filamentous fungal genomes are relatively small and contain a remarkably consistent number of genes. Their genomes range in size from 30 to 100 Mbp and contain 10,000 to 13,000 predicted genes [15]. Their reduced complexity and small size relative to most eukaryotes makes them amenable to assessing the suitability of new sequencing technologies. These technologies have recently been described in the assembly of the filamentous fungus *Sordaria macrospora *[16], which involved a hybrid assembly of Solexa 36-bp reads and 454 sequencing. The objectives of this study were to assemble the genome of *P. teres *f. *teres *based on Solexa sequencing chemistry only, to validate the assembly given the short read lengths (in this study, 75-bp paired ends), and to provide initial characterization of the draft genome. We have complemented the assembly with the first cytogenetic visualization and genome-wide genetic map for this species.

## Results

The genome of *P. teres *f. *teres *isolate 0-1 was sequenced using Illumina's Solexa sequencing platform with paired-end 75-bp reads. The Solexa run in a single flow cell yielded over 833 Mbp of sequence data, or approximately 20 times coverage of the final assembly length. Optimal kmer length in the parallel assembler Assembly By Short Sequences (ABySS) v. 1.0.14 [17] occurred at k = 45 and *n *= 5. This yielded a N_50 _where 50% of the assembly is contained in the largest 408 scaffolds and an L_50 _whereby 50% of the genome is contained in scaffolds of 26,790 bp or more. The total assembly size was 41.95 Mbp. Summary statistics of the assembly are presented in Table 1.

**Table 1:** *Pyrenophora teres *f. *teres *genome assembly key parameters

Parameter	Value
Size (bp)	41,957,260
G + C percentage	48
Predicted protein coding genes ≥100 amino acids	11,089
Predicted protein coding sequences ≥50 amino acids	11,799
Conserved proteins^a^	11,031
Unique hypothetical proteins	766
Percent complete	97.57
Mean gene size (bp)	1411
Mean exon size (bp)	557
Mean number of exons per gene	2.53

^a^Significant at an *e*-value cutoff of ≤10^-5^.

The Solexa sequencing reads that were used for the *P. teres *f. *teres *0-1 genome assembly have been deposited in the NCBI sequence read archive [GenBank: SRA020836]. This whole genome shotgun project assembly has been deposited at DDBJ/EMBL/GenBank under the accession [GenBank: AEEY00000000]. The version described in this paper [GenBank: AEEY01000000] is the first version. Note NCBI does not accept contigs less than 200 bp in whole genome submissions, unless such sequences are important to the assembly, for example, they contribute to scaffolds or are gene coding regions. In addition, all scaffold nucleotide sequences, predicted coding region nucleotide sequences, and translated amino acid sequences are provided in Additional files 1, 2, and 3, respectively.

Both the initial contigs (composed of unpaired reads) and the scaffolds contained a large number of short sequences. In total there were 147,010 initial contigs with an N_50 _of 493 and an L_50 _of 22,178 bp. This compared with a total of 146,737 scaffolds. The majority of initial contigs (140,326 of 147,010) were 200 bp or less, and were shared with the scaffold file. Such short contigs are a result of reads from repetitive regions. In AbySS, where highly similar repetitive regions occur, a 'bubble' removal algorithm simplifies the repeats to a single sequence. Thus, short isolated 'singletons' occur that were not assembled into scaffolds. Gene rich, more complex regions of the genome were represented by 6,684 scaffolds containing over 80% of the assembled sequences.

The assembly contains 11,799 predicted gene models of 50 amino acids or more. Most of the predicted genes (93.5%) were conserved within other species and of these conserved genes, 45.2% showed very high homology with a BLASTP *e*-value of 0. As a further confirmation of the success in capturing gene-rich regions, the percentage of complete genes (genes with defined start and stop codons) was 97.57%.

**Figure 1:**
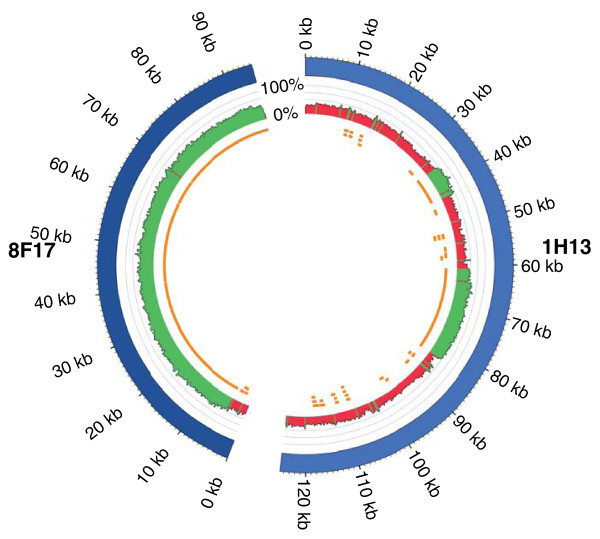
**Comparison of the *P. teres *f. *teres *Solexa assembly with Sanger-sequenced BACs using CIRCOS **[69]. BACs 8F17 and 1H13 are represented in blue. Percent GC is shown in the middle track with regions >40% shown in green and regions <40% shown in red. The inner track shows assembly scaffold BLASTN hits to the BACs.

To validate the assembly over relatively large distances, the assembly was compared to two Sanger sequenced BACs, designated 8F17 and 1H13. Direct BLASTN [18] against assembly scaffolds showed that complex or regions with a high GC content assembled effectively (Figure 1). BAC 1H13 contains several low-complexity regions containing repetitive sequences, in which Solexa reads were over-represented and where only short scaffold assemblies are evident (Additional file 4).

To validate the assembly over short distances of moderately low complexity, and to provide a resource for genetic mapping and genetic diversity studies, we created a set of simple sequence repeats (SSRs). Motif repeats ranged in size from 34 bp with 100% identity and 0% indels to 255 bp with 64% identity and 1% indels. We examined the amplification of a subset (75) of the primer pairs and all gave unambiguous single bands and robust amplification. Primer characteristics and amplicon sizes for the 75 SSRs are provided in Additional file 5. The markers also readily amplified single bands in an isolate of *P. teres *f. *maculata*, albeit with slightly lower efficiency in 20% of the reactions. As a demonstration of their utility, three markers that were polymorphic between *P. teres *f. *teres *and f. *maculata *were used to fingerprint eight randomly selected isolates of each form (Table 2). Markers (ACA)_18_-34213 and (CTG)_19_-61882 were highly polymorphic in *P. teres *f. *teres *and f. *maculata*, respectively, with eight and five alleles. Form-specific diagnostic band sizes are evident from the data, but with overlap in the ranges of allele sizes of each form for (CAT)_13_-49416, and for (ACA)_18_-34213 at 197 bp.

**Table 2:** Inter-form amplification of genome assembly-derived simple sequence repeat markers

	Marker^a^		
	
Isolate	(ACA)_18_-34213	(CAT)_13_-49416	(CTG)_19_-61882
*P. teres *f. *teres*			
Cad 1-3	161	230	177
Cor 2	206	242	180
Cun 1-1	200	230	177
Cun 3-2	215	230	180
NB100	182	230	177
OBR	197	242	180
Stir 9-2	185	228	177
Won 1-1	256	242	177
Number of alleles	8	3	2
			
*P. teres *f. *maculata*			
WAC10721	197	230	196
WAC10981	149	221	189
WAC11177	149	218	189
WAC11185	149	221	189
Cad 6-4	149	221	196
Mur 2	149	221	186
NFR	149	221	199
SG1-1	149	221	190
Number of alleles	2	3	5

Examples of allele sizes from three SSRs are shown for eight randomly selected *P. teres *f. *teres *and *P. teres *f. *maculata *isolates. ^a^Includes SSR motif, template copy number in subscript, and numeric identifier.

In addition to the above assembly validations, we compared 50 randomly selected non-homologous ESTs against the assembly to determine their presence; 49 gave unambiguous matches, with the highest *e*-value cutoff <10^-80^, and one gave no hit. This orphan EST showed no BLASTX similarity to any sequence in GenBank and might be regarded as a library contaminant. Forty-seven (96%) of the remaining ESTs were predicted by GeneMark.

### Electrophoretic and cytological karyotyping of *P*. *teres *f. *teres *

**Figure 2:**
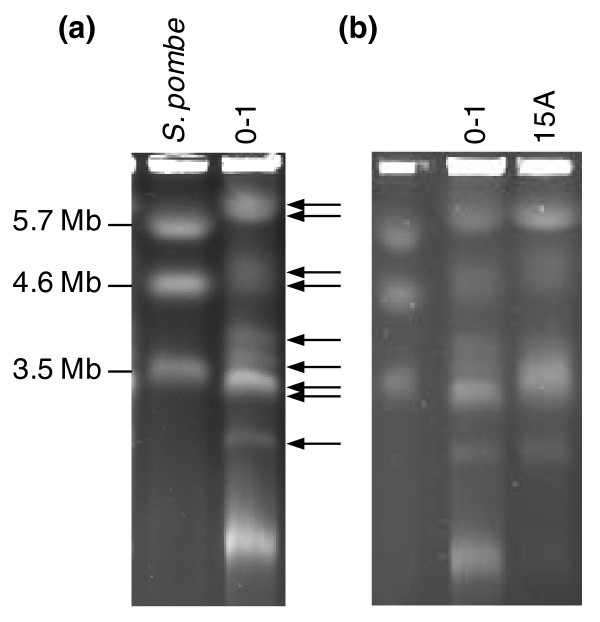
**CHEF (clamped homogenous electric fields) separations of *P. teres *f. *teres *chromosomes**. **(a) **Electro-karyotypes of isolate 0-1 with nine chromosomal bands indicated. **(b) **Chromosome level polymorphisms between isolates 0-1 and 15A.

To estimate the genome size of *P. teres *f. *teres *by pulsed-field gel electrophoresis (PFG), isolate 0-1 was examined and compared to isolate 15A. Isolate 0-1 showed at least seven chromosome bands as indicated in Figure 2, with estimated sizes of 6.0, 4.9, 4.7, 3.9, 3.6, 3.4, and 3 Mbp. The brightness of the band at 6.0 Mbp indicated the presence of at least two chromosomes, and was further resolved into bands of 5.8 and 6.2 Mbp on a second longer electrophoresis run (image not shown). The relative brightness of the 3.4 Mbp band indicates two and possibly three chromosomes are co-migrating. The smallest band visible in Figure 2 is less than 1 Mbp and is most likely mitochondrial DNA. Thus, there is a minimum of nine and as many as eleven chromosomes present in isolate 0-1. This gave an estimated genome size of between 35.5 and 42.3 Mbp. Isolate 15A shows conspicuous differences in the lengths of the chromosomes for intermediate sized bands (greater than 3 Mbp and less than 6 Mbp), and appears to have two bands around 3 Mbp.

**Figure 3:**
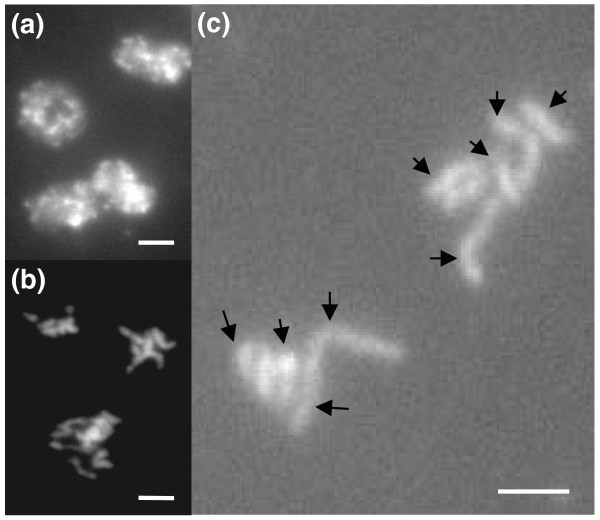
**Visualization of *P. teres *f. *teres *chromosomes using the germ tube burst method (GTBM)**. **(a) **Nuclei at interphase. **(b) **Nuclei at early metaphase. **(c) **Condensed metaphase chromosomes with nine larger chromosomes indicated. Scale bars = 2 μm.

Cytological karyotyping of isolate 0-1 using the germ tube burst method (GTBM) is depicted in Figure 3. Most of the discharged nuclei (above 90%) were observed at interphase (Figure 3a) where the chromosomes exist in the form of chromatin and are enclosed by the nuclear membrane. Of the remaining 10%, most of the chromosomes were either in early metaphase or clumped and entangled together, making it difficult to distinguish chromosomes (Figure 3b). In a few nuclei, condensed metaphase chromosomes were spread out sufficiently and we were able to count at least nine chromosomes (highlighted in Figure 3c). The four largest chromosomes are longer than or equal to 2 μm. The remainder depicted are smaller, but likely to be longer than 1 μm. The four largest chromosomes likely correspond to the four bands shown in PFG electrophoresis that have sizes greater than 3.9 Mbp.

### Gene content

The genome assembly as a whole contains many predicted genes that have been implicated in pathogenicity. Genes encoding efflux pumps have roles in multidrug and fungicide resistance and toxic compound exclusion. For example, the ABC1 transporter in *Magnaporthe grisea *protects the fungus against azole fungicides and the rice phytoalexin sakuranetin [19]. These genes are especially prevalent, with 79 homologues including representatives of the ATP-binding cassette (ABC), major facilitator, and multi antimicrobial extrusion protein superfamilies. Proteins encoded by other notable gene family members are the highly divergent cytochrome P450 s [20], which are involved in mono-oxidation reactions, one member of which has been shown to detoxify the antimicrobial pea compound pisatin [21]; the siderophores, which contribute to iron sequestration and resistance to oxidative and abiotic stresses but which also have essential roles in protection against antimicrobials and formation of infection structures [22,23]; and the tetraspanins, which are required for pathogenicity in several plant pathogenic fungi, one of which is homologous to the newly uncovered Tsp3 family [24].

### Genome-specific expansion of non-orthologous gene families

**Figure 4:**
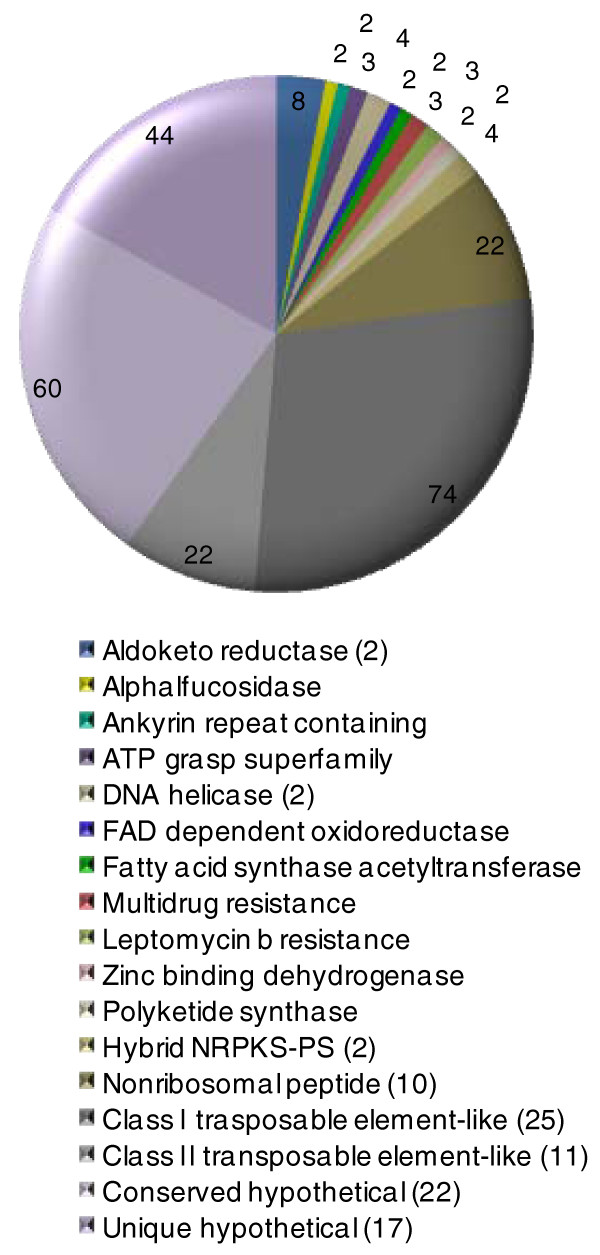
**Expanded *P. teres *f. *teres *gene clusters**. The number of non-orthologous and paralogous genes in each class of genes (as defined by OrthoMCL [25]) is shown at the end of each chart slice and the number of clusters greater than 1 is given in the key.

Cluster analysis of *P. teres *f. *teres *genes in OrthoMCL [25] against the closely related Dothideomycetes species for which genomes and/or ESTs have been made publicly available (*Pyrenophora tritici-repentis*, *Cochliobolus heterostrophus*, *Stagonospora nodorum*, *Leptosphaeria maculans*, *Mycosphaerella graminicola*, together with two *Ascochyta *spp. sequenced in-house, *Ascochyta rabiei *and *Phoma medicaginis *(Ramisah Mod Shah and Angela Williams, personal communication) was used to reveal *P. teres *f. *teres*-specific expansion of gene families. The largest group of these were new members of class I and II transposable elements (Figure 4). Class I transposable elements are retrotransposons that use a RNA intermediate and reverse transcriptase to replicate, while class II transposons use a transposase to excise and reinsert a copy. In total, 36 clusters of new class I and II transposable elements are present in the assembly.

A prominent feature of expanded gene families in *P. teres *f. *teres *is a substantial expansion in specialized multi-functional enzymes known as non-ribosomal peptide synthetases (NRPSs) and polyketide synthases (PKSs) that produce secondary metabolites. The non-orthologous NRPSs are present in 10 clusters of 22 genes. NRPSs catalyze the production of cyclic peptides to form a diverse range of products, including antibiotics and siderophores, and are known to be phytotoxic [26]. Among plant pathogenic Pleosporales fungi, HC toxin from *Cochliobolus carbonum *[27] and AM toxin from *Alternaria alternata *[28] are notable examples. Also evident are hybrid NRPS-PKSs [29] in two clusters of four genes. PKSs produce polyketides in a manner similar to fatty acid biosynthesis. In fungi, better known polyketides are the mycotoxins fumonisin and autofusarin, and the phytotoxin cercosporin [30]. Hybrid NRPS-PKSs occur where PKS and NRPS modules coexist and add to the complexity of secondary metabolites. Most of the remaining non-orthologous gene clusters include homologues to genes involved with secondary metabolism and signaling. Investigations into the functional significance of these genes may provide new insights into the requirements of this pathogen. Also present are six non-orthologous genes encoding antibiotic and multi-drug resistance proteins that may have a role against toxic plant compounds. Indeed, the *P. teres *f. *teres *assembly as a whole contains ten genes with homology to ABC drug transporters.

### Secreted proteins

Comparisons between plant pathogenic ascomycetes *S. nodorum *and *M. grisea *with the saprophyte *Neurospora crassa *[31,32] have both shown the expansion of secreted gene families consistent with their roles as plant pathogens. *P. teres *f. *teres *contains a large number of genes (1,031) predicted to be secreted by both WolfPSORT [33] and SignalP [34]. A significant proportion of these genes in *P. teres *f. *teres *(85%) are homologous with *P. tritici repentis*, as might be expected given their close phylogenetic relationship. This contrasts with 54% of the predicted genes in *S. nodorum *for which no phylogenetically close relative was sequenced [32]. Of the remaining genes, a small number (1.6%) show strongest homology to species outside the Pleosporales, while 6% are unique to *P. teres *f. *teres *isolate 0-1 with no functional annotation. These genes may include genes that have been laterally transferred.

In Blast2GO [35,36], 61.6% of the predicted genes were annotated with Gene Ontology (GO) terms. GO annotations are limited to well characterized genes but they do provide a useful overview. A large proportion of predicted genes encode proteins associated with plant cell wall and cutin degradation, presumably to degrade plant tissue during necrotrophic growth. Most are protein and carbohydrate hydrolases, together with carbohydrate binding proteins that target various polysaccharides (Table 3). For example, there are nine and seven predicted gene products with homology to cellulose binding proteins and cellulases, respectively, and five and four predicted gene products with homology to cutin binding proteins and cutinases, respectively. Predicted proteins annotated with the GO term 'pathogenesis' include homologues of glycosyl hydrolases, cutinase precursors, surface antigens, and a monoxygenase related to maackiain detoxification protein from *Nectria haematococca *[37].

**Table 3:** Common GO terms associated with genes predicted to be secreted

GO identifier	Description	Number of genes
Biological process		
GO:0006508	Proteolysis	42
GO:0055114	Oxidation reduction	25
GO:0043581	Mycelium development	23
GO:0051591	Response to cAMP	16
GO:0045493	Xylan catabolic process	14
GO:0009405	Pathogenesis	9
GO:0034645	Macromolecule biosynthesis	8
GO:0044248	Cellular catabolic process	7
GO:0021700	Developmental maturation	7
GO:0006139	Nucleic acid metabolism	7
GO:0050794	Regulation of cellular process	7
GO:0006629	Lipid metabolic process	7
GO:0019222	Metabolic regulation	6
GO:0016998	Cell wall catabolic process	6
GO:0034641	Nitrogen metabolism	6
GO:0030245	Cellulose catabolic process	6
GO:0006032	Chitin catabolic process	6
GO:0006979	Response to oxidative stress	6
GO:0009847	Spore germination	6
GO:0007154	Cell communication	5
GO:0006464	Protein modification process	5
		
Molecular function		
GO:0016787	Hydrolase activity	193
GO:0043167	Ion binding	84
GO:0016491	Oxidoreductase activity	73
GO:0048037	Cofactor binding	36
GO:0000166	Nucleotide binding	36
GO:0030246	Carbohydrate binding	26
GO:0046906	Tetrapyrrole binding	16
GO:0001871	Pattern binding	14
GO:0016740	Transferase activity	13
GO:0016829	Lyase activity	9
GO:0005515	Protein binding	6
GO:0016874	Ligase activity	6
GO:0016853	Isomerase activity	6

Terms are filtered for ≥5 members; molecular function GO terms are limited to GO term level 3.

### Marker development and linkage map construction

A total of 279 amplified fragment length polymorphisms (AFLPs) were generated that were polymorphic between the mapping population parents 15A and 0-1 using 96 primer combinations of 8 *Mse*I primers and 12 *Eco*RI primers (Additional file 6). On average, each pair produced approximately three polymorphic AFLPs. We identified a total of 68 polymorphic SSRs for genetic mapping; 44 from the genome assembly sequence, 20 from sequence tagged microsatellite site (STMS) markers [38], and 4 from ESTs (Additional file 5). In addition to AFLPs and SSRs, five random amplified polymorphic DNA markers associated with *AvrHar *[39] and the mating type locus were genotyped across 78 progeny from the 15A × 0-1 cross. All markers were tested for segregation ratio distortion; 69 (19%) were significantly different from the expected 1:1 ratio at *P *= 0.05, of which 32 were distorted at *P *= 0.01.

**Figure 5:**
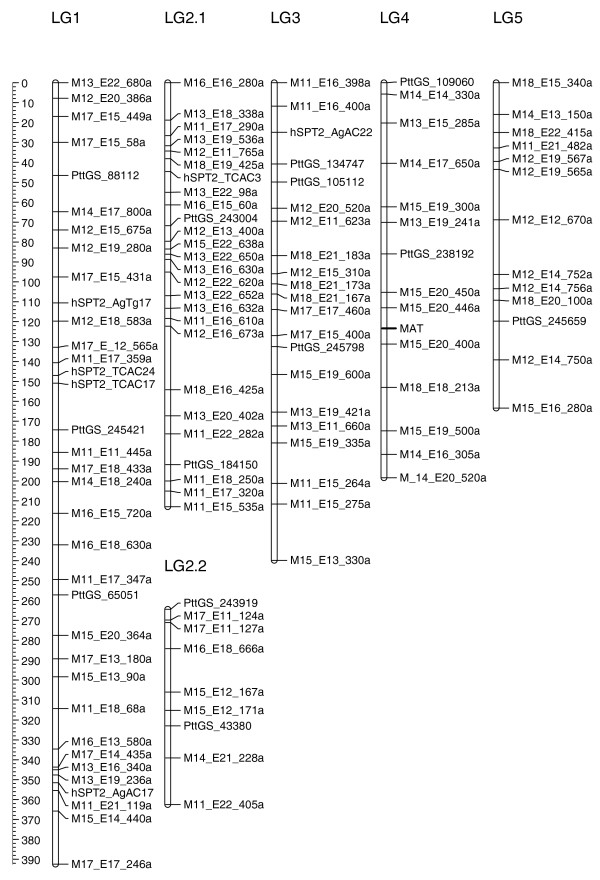
**Genetic linkage map of *P. teres *f. *teres***. Linkage groups are drawn with genetic distance in cM on the scale bar to the left and are ordered according to their genetic length. AFLP markers are indicated by the *Mse*I (M) and *Eco*RI (E) primer combination (Additional file 6), followed by the size of the marker. SSR markers were developed from three sources: ESTs, STMSs and the genome assembly, prefixed PtESTSSR_, hSPT2_, and PttGS_, respectively. The mating type locus (MAT) is depicted in bold on linkage group 4.

**Figure 6:**
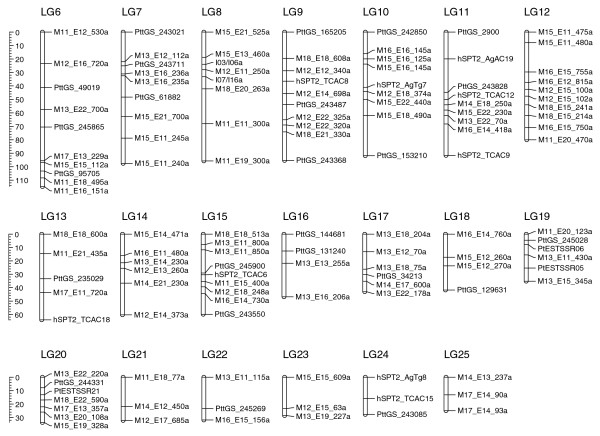
**Genetic linkage map of *P. teres *f. *teres *continued from Figure 5**. Linkage groups are drawn with genetic distance in cM on the scale bar to the left and are ordered according to their genetic length.

The genetic map was initially constructed with a total of 354 markers composed of 279 AFLPs, 68 SSRs, 5 random amplified polymorphic DNA markers, and a single mating type locus marker. The markers were first assigned into groups using a minimum LOD (logarithm of the odds) threshold of 5.0 and a maximum θ = 0.3. We excluded 111 markers from the map because they had a LOD <3 by RIPPLE in MAPMAKER [40]. The final genetic map was composed of 243 markers in 25 linkage groups, with each linkage group having at least 3 markers. The map spans 2,477.7 cM in length, with an average marker density of approximately one marker per ten centiMorgans (Figures 5 and 6). Individual linkage groups ranged from 24.9 cM (LG25) to 392.0 cM (LG1), with 3 and 35 markers, respectively. Three of the linkage groups had a genetic distance greater than 200 cM and 10 linkage groups had genetic distances of less than 50 cM, leaving 12 medium-sized linkage groups ranging between 50 and 200 cM. Other than a 30-cM gap on LG2.1, the markers are fairly evenly distributed on the linkage groups without obvious clustering. Linkage groups 2.1 and 2.2 are provisionally aligned together in Figure 5 as they may represent a single linkage group. This association is based on forming a single linkage group at LOD = 2, and by comparative mapping of SSR scaffold sequences with the *P. tritici-repentis *assembly (data not shown). The mating type locus mapped to linkage group LG4, and except for six of the small linkage groups, each linkage group has at least one SSR marker, which may allow comparisons to closely related genome sequences.

## Discussion

This is the first wholly Illumina-based assembly of an ascomycete genome and the third assembly to be reported for a necrotrophic plant pathogenic ascomycete [31,32]. As might be expected, the *P. teres *f. *teres *genome assembly demonstrates that the short paired-end reads can be used to effectively capture higher complexity gene-containing regions. The assembly was validated by comparison to BAC sequences, ESTs and by direct amplification of predicted sequences across SSRs. Based on the published assemblies for the phytopathogens *M. grisea *and *S. nodorum *[31,32], the number of predicted genes in *P. teres *f. *teres *is similar (11,089 versus 11,109 and 10,762, for genes larger than 100 amino acids or *S. nodorum *version 2 gene models, respectively). Gene prediction algorithms, even when trained on ESTs from the species in question, are unlikely to correctly predict all coding regions in more complex genomes, and in some instances require further corroborating data from approaches such as proteomics and mass-spectrometry [41]. Thus, the true number of genes may be less dependent on the assembly *per se *and gene models may be further adjusted, concatenated or introduced.

The inevitable corollary of an assembly based on short paired-end reads is that low-complexity regions (containing low GC content, simple microsatellites and repetitive DNA) are under-represented. As a consequence, the assembly is composed of a large number of singleton contigs that are inappropriate for estimating the genomic proportions of such regions. To support the minimum estimate of the genome size based on the assembly, and to provide basic information on chromosome composition, we conducted PFG and GTBM karyotyping. From the PFG results, we concluded that *P. teres *f. *teres *most likely contains a minimum of 9 chromosomes but with band intensities suggesting 11 chromosomes is possible. This provided an estimated genome size of at least 35.5 Mbp and an upper value of 42.3 Mbp. Clumping and co-migration of bands is a common phenomenon in PFG, as shown, for example, by Eusebio-Cope *et al*. [42]. Resolution of co-migrating bands requires techniques such as Southern blotting [43] and fluorescence *in situ *hybridization [44] for accurate discrimination. However, the cytological karyotyping correlated with the PFG results in depicting at least nine chromosomes. An upper estimate of nine chromosomes was postulated for *P. teres *by Aragona *et al*. [45], although that study did not identify which *P. teres *form was examined, and the technique used gave poor resolution of bands between 4.5 and >6 Mbp. Overall, the total assembly size in this study correlates with the higher estimate by elecrophoretic karyotyping and indicates a genome of at least 42 Mbp. This is somewhat larger than the Pleosporales assemblies reported to date for *Cochliobolus heterostrophus *(34.9 Mbp; Joint Genome Institute), *P. tritici-repentis *(37.8 Mbp; NCBI) and *S. nodorum *(37.1 Mbp [32]).

An expansion in genome size compared to other Pleosporales might be explained by the presence in the assembly of new classes of transposable elements and large numbers of novel repeats (over 60, although these data are incomplete due to poor assembly of degraded regions and therefore have not been shown). These in turn may also explain the large PFG chromosomal level polymorphisms between the two isolates examined here and the relatively large genetic map. Chromosomal level polymorphisms are a feature of some ascomycetes [46]. Among plant pathogenic fungi, there is growing evidence that host-specificity genes and effectors are located in or next to transposon-rich regions [31,47]. This provides opportunities for horizontal acquisition, duplication and further diversification to generate new, species-specific genetic diversity or, where they are recognized as an avirulence gene, to be lost, a process that may also aid host range expansion. The contribution of transposons in *P. teres *f. *teres *pathogenicity has yet to be determined, although we have preliminary data showing that the avirulence gene *AvrHar *is associated with transposon repeats on the second largest chromosome. There is no evidence in *P. teres *f. *teres *for small chromosomes <2 Mbp, as in *N. haematococca *and *A. alternate*, where they confer host-specific virulence [48,49], and in *Fusarium oxysporum*, where they have been demonstrated to be mobile genetic elements conferring virulence to non-pathogenic strains [50].

The analysis of the gene content of the genome assembly shows that it shares many of the characteristics of similar plant pathogenic fungi, and strong homology to most genes from *P. tritici-repentis*. These include highly diverse proteins involved in host contact, signal transduction, secondary metabolite production and pathogenesis. Secreted proteins are of particular interest to plant pathologists since they represent the key interface of host-pathogen interactions, notably avirulence proteins and effectors. These are key components of inducing disease resistance and promoting disease, while expressed effector proteins offer tangible discriminating resistance assay tools in a variety of breeding programs. This is because fungal necrotrophic disease is the sum of the contribution of individual effectors [51,52] and single, purified effectors give a qualitative response when infiltrated into leaves. However, effector genes often encode small, cysteine-rich proteins with little or no orthology to known genes. Examples include *Avr2 *and *Avr4 *in *Cladosporium fulvum*, *Avr3 *in *F. oxysporum *(reviewed in [53]), *ToxA *and *ToxB *in *P. tritici repentis *[54,55] and *SnToxA *and *SnTox3 *in *S. nodorum *[56,57]. Identifying candidate effectors in the genome assembly in conjunction with genetic mapping, functional studies and proteomic approaches will in future aid their isolation.

We provide the first genetic linkage map of *P. teres *f. *teres*. The total length is nearly 2,500 cM, longer than that reported for other ascomycete fungal pathogens; 1,216 cM for *M. graminicola *[58], 1,329 cM for *Cochliobus sativus *[59], and 900 cM for *M. grisea *[60]. However, a genetic map of 359 loci for the powdery mildew fungus *Blumeria graminis *f. sp. *hordei*, an obligate biotrophic pathogen of barley, covered 2,114 cM [61]. The length of the genetic map of *P. teres *f. *teres *may be a function of the relatively large genome size and the presence of large numbers of recombinogenic repetitive elements. This is paralleled by a greater number of linkage groups (25) compared to the estimated number of chromosomes that may also be suggestive of interspersed tracts of repetitive DNA.

The genetic map and karyotyping data will be instrumental in a final assembly of the *P. teres *f. *teres *genome, as they will allow scaffolds to be orientated and tiled onto linkage groups. A combination of the genome assembly and the genetic map provides an invaluable resource to identify potential effector candidate genes from phytotoxic protein fractions in conjunction with mass spectrometry peptide analysis. Genetically characterized SSRs provided in this study will also provide an important resource for the community in comparative mapping, gene-flow and genetic diversity studies. Further validation, assembly of low-complexity sequence regions, and genome annotation are now underway using proteomic approaches and 454 pyrosequencing. The priority now is to fully understand the mechanism of pathogenicity in *P. teres *f. *teres *in order to achieve a solution to control this pathogen.

## Conclusions

This study demonstrates that the successful assembly of more complex and gene-rich regions of a filamentous fungus is possible using paired-end Solexa sequencing. The approach provides a cost-effective means of directly generating marker resources that would previously have been prohibitively expensive with modest research funding. At 42 Mbp or more, the genome of *P. teres *f. *teres *0-1 is larger by comparison to closely related Pleosporales members, and has a correspondingly large genetic map. The genome is dynamic, in that different isolates show obvious chromosomal level differences, while fractionated linkage groups and the length of the genetic map also suggest an abundance of repetitive DNA. In common with other plant pathogens, *P. teres *f. *teres *contains a rich diversity of predicted genes, notably protein and carbohydrate hydrolases, efflux pumps, cytochrome P450 genes, siderophores, tetraspanins, non-ribosomal peptide synthetases and polyketide synthases, and a complex secretome that can be attributed to its lifestyle. Non-ribosomal peptide synthetases and efflux pumps in particular appear to have undergone a *P. teres *f. *teres*-specific expansion of non-othologous gene families. The assembly presented provides researchers with an excellent resource to further examine net blotch pathogenicity and plant-microbe interactions in general.

## Materials and methods

### Origin of *P. teres *isolates

The NFNB isolate sequenced in this study, 0-1, was originally collected in Ontario, Canada [39]. Isolate 15A (10-15-19), the opposite parental isolate used to develop a mapping population, was collected from Solano County, California [62]. The remaining NFNB isolates (Cad 1-3, Cor 2, Cun 1-1, Cun 3-2, NB100, OBR, Stir 9-2, and Won 1-1) were collected in Western Australia by S Ellwood in the 2009 barley growing season. SFNB isolates WAC10721, WAC10981, WAC11177, and WAC11185 were obtained from the Department of Agriculture and Food, Western Australia (3, Baron Hay Court, South Perth, Western Australia 6151); isolates Cad 6-4, Mur 2, NFR, and SG1-1 were collected in Western Australia by S Ellwood during 2009.

### Electrophoretic and cytological karyotyping

#### Protoplasting and pulsed-field gel electrophoresis

Chromosome size and number were analyzed for North American NFNB isolates; 0-1 and 15A, previously used to develop a genetic cross for identifying avirulence genes [39,63]. Fungal protoplasts were prepared using a protocol established for *S. nodorum *as described by Liu *et al*. [56] with some modifications. Briefly, conidia were harvested from 7-day fungal cultures and inoculated into 60 ml liquid Fries medium in 250 ml Erlenmeyer flasks. After growth at 27°C in a shaker (100 rpm) for 48 h, the fungal tissue was then homogenized in a Waring blender and re-inoculated into 200 ml liquid Fries medium in 500 ml Erlenmeyer flasks. The fungus was grown under the same growth conditions for 24 h. Mycelium was harvested by filtering through two layers of Miracloth, washed thoroughly with water and finally with mycelial wash solution (MWS: 0.7 M KCl and 10 mM CaCl_2_). Around 2 g (wet weight) of mycelial tissue was then transferred into a Petri dish (100 × 20 mm) containing 40 ml filter-sterilized protoplasting solution containing 40 mg/ml β-d-glucanase, 0.8 mg/ml chitinase, and 5 mg/ml driselase (Interspex Product Inc., San Mateo, CA, USA) in MWS. The Petri dish was shaken at 70 rpm at 28°C for at least 5 h. Protoplasts were filtered through four layers of Miracloth and pelleted by centrifugation at 2,000 × *g *for 5 minutes at room temperature, followed by another wash with MWS and pelleting. Protoplasts were resuspended in MWS to a final concentration of 2 × 10^8 ^protoplasts/ml and mixed with an equal volume of 2% low melting temperature agarose (Bio-Rad Laboratories, Hercules, CA, USA) dissolved in MWS. Agarose plugs were made by pipetting 80 μl of the mixture into plug molds (Bio-Rad Laboratories). Once solidified, plugs were placed in 20 ml Proteinase K reaction buffer containing 100 mM EDTA (pH 8.0), 1% N-lauroyl sarcosine, 0.2% sodium deoxycholate and 1 mg/ml Proteinase K (USBiological, Swampscott, MA, USA) at 50°C for 24 h. Plugs were washed four times in 10 mM Tris pH 8.0 and 50 mM EDTA for 1 h with gentle agitation, then stored in 0.5 M EDTA (pH 8.0) at 4°C. PFG was performed on a Bio-Rad CHEF Mapper system. Separation of chromosomes in the 1 to 6 Mb range was carried out in 1.0× TAE at 14°C using 0.8% Low EEO agarose gel (USBiological). Run time was 72 h at 2 V/cm (70 V) with a 20- to 40-minute switch time ramp at an angle of 106°.

#### Spore germination and germ tube burst cytological karyotyping

Conidia were washed with water from 7-day cultures grown on V8 potato dextrose agar (V8PDA) plates, filtered through two layers of miracloth and centrifuged at 3,000 × *g *for 5 minutes. Conidia were washed twice with potato dextrose broth and re-suspended in this with a final concentration of 4 × 10^5 ^spores/ml. Approximately 400 μl of spore suspension was placed onto slides coated with poly-L-lysine (Sigma-Aldrich Corp., St Louis, MO, USA) and covered by a 22 × 40 mm piece of parafilm to keep moist. All slides were kept in a sealed plastic box at room temperature for 3 h, and then moved to the fridge for cold treatment overnight. Slides were dipped in H_2_O to carefully remove the covers and then placed in a methanol/acetic acid (22:3) solution overnight to fix fungal tissue. The slides were flame dried to burst cells and release chromosomes. Slides were stained for 5 minutes in the dark with 1 μg/ml 4',6-diamidino-2-phenylindole (DAPI; Sigma-Aldrich) and 1 μg/ml Flourescent Brightener 28 (Sigma-Aldrich) in anti-fade mounting solution. Slides were examined and photographed using a Zeiss Axioplan 2 epiflourescent microscope.

### Genome sequence acquisition

#### Whole shotgun genome sequencing

DNA of *P. teres *f. *teres *isolate 0-1 was extracted using a Biosprint DNA Plant Kit and a BioSprint 15 automated workstation (Qiagen, Hilden, Germany). Genomic sequencing was performed on a Solexa sequencing platform at the Allan Wilson Centre (Massey University, Palmerston North, New Zealand). DNA preparation, cluster formation, primer hybridization and DNA amplification reactions were according to the manufacturer's recommended protocol [64]. DNA sequencing was performed using 75-bp paired-end reads of randomly sheared 200-bp fragments in a single flow cell. Data were pre-filtered in Illumina's Pipeline v.1.4 and IPAR v.1.3. Reads failing a 'chastity' filter of 0.6 were discarded. The steps described below for genome scaffold assembly, annotation and analysis were performed on the iVEC advanced computing facilities [65].

#### Paired-end scaffold assemblies

Single (split pairs) and paired-end reads were assembled using ABySS v.1.0.14 [17]. In addition to the read filtering described above, ABySS removes reads containing ambiguous characters (Ns). The optimal sequence kmer (overlap) length was determined by incrementally adjusting the kmer length by 4 bp and graphing the number of contigs against L_50 _for a given kmer length. The optimal kmer length occurred where N_50 _was minimal and L_50 _was largest as visualized by R [66]. N_50 _is a weighted median statistic such that 50% of the entire assembly is contained in the number of contigs or scaffolds equal to or greater than this value, while L_50 _is the length of the scaffold that separates the half of the assembled genome from the remainder of smaller scaffolds, if the sequences are ordered by size.

#### Annotation and analysis

Protein coding sequences were identified with GeneMark-ES v.2 [67]. GeneMark uses a self-training algorithm optimized for features of fungal gene organization by incorporating an enhanced intron submodel to accommodate sequences with and without branch point sites. GeneMark compares favorably with the accuracy of gene finders that employ supervised training based on cDNA sequences.

Annotation of predicted proteins was conducted with the following tools. A mirror of the NCBI database at iVEC, together with publicly available fungal protein sequence files not present at NCBI, was interrogated by BLASTP [18]. Blast2GO v.2.4.2 [35,36], which incorporates GO, KEGG maps, InterPro and Enzyme Codes was used with default parameters for functional annotation. *De novo *annotation of PFAM domains was performed using HMMER v.2.3.2 [68]. HMMER searches for homologues of protein sequences and implements methods using probabilistic models called 'profile hidden Markov models'. To detect orthologous genes, we used OrthoMCL [25] by BLAST to the NCBI non-redundant database with an *e*-value cutoff of ≤10^-5^. OrthoMCL is a genome-scale algorithm for grouping protein sequences between species based on BLAST similarity that was used to identify species-specific expanded gene families. Subcellular localization of proteins and secretion signals were identified with Wolf PSort [33] and SignalP v.3.0 [34] using default parameters and selection of the appropriate organism type.

### Genome assembly validation

#### Assembly comparison with Sanger-sequenced BACs

To validate the assembly over a larger scale, BLASTN [18] was used to compare the assembly against two NFNB 0-1 BACs, designated 8F17 and 1H13, sequenced and assembled by The Genome Center (Washington University, St Louis, MO, USA). The data were visualized with CIRCOS [69]. To establish if all regions of the BACs were covered by Solexa sequencing, raw reads were mapped to the BACs with the Burrows-Wheeler Aligner [70] and visualized using R and the ggplot2 package [66,71].

#### SSR primer design and PCR amplification

Short tandem repeats or SSRs (also known as microsatellites) were identified by scanning the genome assembly with Tandem Repeat Finder v.4 [72] for a minimum of ten tandem repeats from 2 to 7 bp. Primers were designed using Primer3 [73] using parameters designed to minimize secondary structures, with a GC content >40%, and an optimum melting temperature of 58 to 60°C, for amplicons in a size range of 150 to 400 bp. The primers were assayed using single-spored *P. teres *isolates collected from different sites in Western Australia. DNA extraction and PCR amplification using the Multiplex Ready Technique were performed as described previously [74,75]. Allele sizing was performed using GeneMapper v.3.7 (Applied Biosystems, Foster City, CA, USA).

#### EST library preparation, sequencing, and assembly comparison

Total RNA was extracted from isolate 0-1 using fungal mycelium tissue grown in liquid Fries medium for 4 days. The RNA was extracted with TRIZOL (Invitrogen, Carlsbad, CA, USA) following the manufacturer's instructions. EST library construction and sequencing was conducted by The Genome Center. To investigate the presence of ESTs in the assembly and the efficiency of GeneMark predictions, unique EST sequences were BLASTN searched against the assembly. BLASTN hits were then compared against the location of GeneMark predicted coding regions with BEDtools [76].

### Marker development and genetic linkage map construction

Lai *et al*. [63] used a subset of AFLPs to identify markers associated with fungal avirulence on the barley lines 'Harbin' and 'Prato' on two linkage groups. That study used a segregating population of 78 progeny from a cross between NFNB isolates 15A and 0-1. The AFLP markers were generated based on the technique of Vos *et al*. [77] and employed 96 primer combinations containing *Eco*RI and *Mse*I restriction sites. In this study, all available AFLPs from the 96 primer combinations were used to develop a comprehensive genetic map (Additional file 6). In addition, we incorporated polymorphic STMSs developed from microsatellite libraries by Keiper *et al*. [38], together with SSRs from EST sequences and the genome assembly herein. SSR PCR amplification and population genotyping were performed as described previously [38,78,79]. In addition, the mating type locus was assayed using primers Pt5 and Pt7 that amplify the *P. teres *HMG box [80].

Linkage map construction was performed with MAPMAKER v.2.0 for Macintosh as described by Liu *et al*. [78]. A minimum LOD value of 5.0 and a maximum θ = 0.3 were used to establish the linkage groups. For each linkage group, the most plausible order of markers was determined using commands 'FIRST ORDER' and 'RIPPLE', and markers with low confidence (LOD <3.0 for RIPPLE) were excluded from the map. All markers were tested for fitness of a 1:1 segregation ratio using *Qgene *[81]. The genetic map was drawn with the software program MapChart v.2.1 [82].

## Abbreviations

ABC, ATP-binding cassette; AFLP, amplified fragment length polymorphism; BAC, bacterial artificial chromosome; bp, base pair; DDBJ, DNA Data Bank of Japan; EST, expressed sequence tag; GO, Gene Ontology; GTBM, germ tube burst method; LOD, logarithm of the odds; MWS, mycelial wash solution; NCBI, National Center for Biotechnology Information; NFNB, net form of net blotch; NRPS, non-ribosomal peptide synthetase; PFG, pulsed-field gel electrophoresis; PKS, polyketide synthase; SFNB, spot form of net blotch; SSR, simple sequence repeat; STMS, sequence-tagged microsatellite site.

## Authors' contributions

SRE analyzed the data, wrote the manuscript, and performed initial laboratory SSR genetic marker validation. RAS provided informatics expertise in software implementation, scripting and primary data analysis (including for genome assembly, gene prediction, annotation of predicted peptides, and homology searches). JKH provided scripts to facilitate SSR design and data elucidation. CSM assisted with comparisons of predicted *Pyrenophora *proteins. ZL screened for polymorphic SSR markers on genetic mapping population parents and conducted genotyping, genetic map construction, and electrophoretic karyotyping. FK contributed STMS markers. ZL undertook the AFLP genotyping and the cytological karyotyping. RPO and TLF contributed to the design of the project and provided assistance in finalizing the manuscript prior to publication.

## Supplementary Material

Additional file 1 ***P. teres *f. *teres *isolate 0-1 scaffold assembly nucleotide sequences**. Click here for file

Additional file 2 ***P. teres *f. *teres *isolate 0-1 predicted coding region nucleotide sequences**. Click here for file

Additional file 3 ***P. teres *f. *teres *isolate 0-1 predicted coding region translated amino acid sequences**. Click here for file

Additional file 4 **Solexa read coverage of BACs 1H13 and 8F17**. Click here for file

Additional file 5 **Characteristics of 75 genome assembly-derived SSRs and those polymorphic SSRs used in the *P. teres *f. *teres *01 × 15A genetic map construction**. Click here for file

Additional file 6 **AFLP di-nucleotide selective primer extensions and their codes**. Click here for file
