# Supplementary material for: A first genome assembly of the barley fungal pathogen Pyrenophora teres f. teres
Source: Genome Biol. 2010 Nov 10;11(11):R109. doi: 10.1186/gb-2010-11-11-r109 (PMC3156948; doi:10.1186/gb-2010-11-11-r109)
Supplement: Additional file 4 — Solexa read coverage of BACs 1H13 and 8F17. [file gb-2010-11-11-r109-S4.docx]

Solexa read coverage of BACs 1H13 and 8F17. GC percentage across the BACs is represented by the black line and the relative number of read hits (from 0 to 1000) by blue vertical bars.


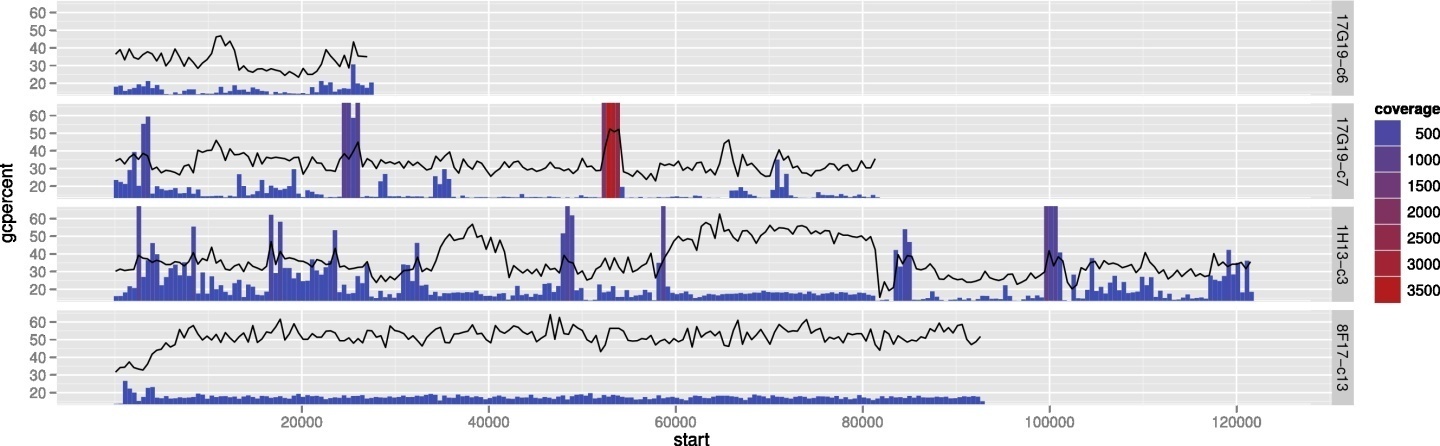


GC (%)

Distance (kbp)
