# Supplementary material for: A first genome assembly of the barley fungal pathogen Pyrenophora teres f. teres
Source: Genome Biol. 2010 Nov 10;11(11):R109. doi: 10.1186/gb-2010-11-11-r109 (PMC3156948; doi:10.1186/gb-2010-11-11-r109)
Supplement: Additional file 6 — AFLP di-nucleotide selective primer extensions and their codes. [file gb-2010-11-11-r109-S6.docx]

AFLP di-nucleotide selective primer extensions and their codes.

| EcoRI | | Mse I | |
| --- | --- | --- | --- |
| 5'-GACTGCGTACCAATTCNN-3' | | 5’-GATGAGTCCTGAGTAANN-3' | |
|  |  |  |  |
| E11 | AA-3’ | M11 | AA-3’ |
| E12 | AC-3’ | M12 | AC-3’ |
| E13 | AG-3’ | M13 | AG-3’ |
| E14 | AT-3’ | M14 | AT-3’ |
| E15 | CA-3’ | M15 | CA-3’ |
| E16 | CC-3’ | M16 | CC-3’ |
| E17 | CG-3’ | M17 | CG-3’ |
| E18 | CT-3’ | M18 | CT-3’ |
| E19 | GA-3’ |  |  |
| E20 | GC-3’ |  |  |
| E21 | GG-3’ |  |  |
| E22 | GT-3’ |  |  |
